# A monthly gridded burned area database of national wildland fire data

**DOI:** 10.1038/s41597-024-03141-2

**Published:** 2024-04-08

**Authors:** Andrina Gincheva, Juli G. Pausas, Andrew Edwards, Antonello Provenzale, Artemi Cerdà, Chelene Hanes, Dominic Royé, Emilio Chuvieco, Florent Mouillot, Gabriele Vissio, Jesús Rodrigo, Joaquin Bedía, John T. Abatzoglou, José María Senciales González, Karen C. Short, Mara Baudena, Maria Carmen Llasat, Marta Magnani, Matthias M. Boer, Mauro E. González, Miguel Ángel Torres-Vázquez, Paolo Fiorucci, Peter Jacklyn, Renata Libonati, Ricardo M. Trigo, Sixto Herrera, Sonia Jerez, Xianli Wang, Marco Turco

**Affiliations:** 1 Regional Atmospheric Modelling (MAR) Group, Department of Physics, Regional Campus of International Excellence Campus Mare Nostrum (CEIR), University of Murcia, Murcia, Spain; 2 Centro de Investigaciones sobre Desertificación, Spanish National Research Council (CIDE-CSIC), Valencia, Spain; 3 Darwin Centre for Bushfire Research, Charles Darwin University, Darwin, Northern Territory Australia; 4 Institute of Geosciences and Earth Resources - National Research Council of Italy (CNR-IGG), Turin, Italy; 5 National Biodiversity Future Center, Palermo, Italy; 6 Soil Erosion and Degradation Research Group, Department of Geography, Valencia University, Valencia, Spain; 7 Great Lakes Forestry Centre, Canadian Forest Service, Natural Resources Canada, Sault Ste. Marie, Ontario, Canada; 8 Climate Research Foundation (FIC), Madrid, Spain; 9 Universidad de Alcalá, Environmental Remote Sensing Research Group, Department of Geology, Geography and the Environment, Alcalá de Henares, Spain; 10 UMR CEFE, Université de Montpellier, CNRS, EPHE, IRD, Montpellier, France; 11 Departamento de Análisis Geográfico Regional y Geografía Física, Facultad de Filosofía y Letras, Campus Universitario de Cartuja, Universidad de Granada, Granada, Spain; 12 Departamento Matemática Aplicada y Ciencias de la Computación (MACC), Universidad de Cantabria, Santander, Spain; 13 Grupo de Meteorología y Computación, Universidad de Cantabria, Unidad Asociada al CSIC, Santander, Spain; 14 Management of Complex Systems, University of California, Merced, USA; 15 Department of Geography, University of Málaga, Málaga, Spain; 16 Department of Agriculture, Forest Service, Missoula Fire Sciences Laboratory, Missoula, Montana USA; 17 Institute of Atmospheric Sciences and Climate, National Research Council of Italy (CNR- ISAC), Torino, Italy; 18 GAMA, Department of Applied Physics, Universitat de Barcelona, Barcelona, Spain; 19 Hawkesbury Institute for the Environment, Western Sydney University, Penrith, NSW Australia; 20 NSW Bushfire and Natural Hazards Research Centre, Richmond, NSW Australia; 21 Instituto de Conservación, Biodiversidad y Territorio, Facultad de Ciencias Forestales y Recursos Naturales, Universidad Austral de Chile, Valdivia, Chile; 22 Center for Climate and Resilience Research (CR2), Santiago, Chile; 23 CIMA Research Foundation, Savona, Italy; 24 Instituto de Geociências, Universidade Federal do Rio de Janeiro, Rio de Janeiro, Brazil; 25 Instituto Dom Luiz (IDL), Faculdade de Ciências, Universidade de Lisboa, Lisbon, Portugal; 26 Applied Mathematics and Computer Science Department Universidad de Cantabria, Santander, Spain; 27 Northern Forestry Centre, Canadian Forest Service, Natural Resources Canada, Edmonton, AB Canada

**Keywords:** Environmental impact, Natural hazards

## Abstract

We assembled the first gridded burned area (BA) database of national wildfire data (ONFIRE), a comprehensive and integrated resource for researchers, non-government organisations, and government agencies analysing wildfires in various regions of the Earth. We extracted and harmonised records from different regions and sources using open and reproducible methods, providing data in a common framework for the whole period available (starting from 1950 in Australia, 1959 in Canada, 1985 in Chile, 1980 in Europe, and 1984 in the United States) up to 2021 on a common 1° × 1° grid. The data originate from national agencies (often, ground mapping), thus representing the best local expert knowledge. Key opportunities and limits in using this dataset are discussed as well as possible future expansions of this open-source approach that should be explored. This dataset complements existing gridded BA data based on remote sensing and offers a valuable opportunity to better understand and assess fire regime changes, and their drivers, in these regions. The ONFIRE database can be freely accessed at https://zenodo.org/record/8289245.

## Background & Summary

Humans have coexisted with wildfires for millennia, and fire has played a relevant role in maintaining ecosystem services for societies worldwide^1,2^. However, losses of lives and goods (i.e. natural resources and built assets) are also associated with wildfires, leading to growing concerns about increased fire activity in response to climate change^3^. The study of fire regime changes and their underlying causes is crucial for the development of effective fire management strategies and future prevention plans in the face of ongoing global changes^4,5^. Despite recent advancements in fire data availability and research efforts, much remains to be learned regarding fire trends and drivers^4,6–8^.

A growing body of work uses fire statistical estimates based on satellite products^9–12^; however, only recent decades (i.e., since the early 2000s^13,14^) have afforded the mature technology to produce the first global Burned Area (BA) datasets^14^. Moreover, remote sensing-based BA estimates have some limitations, including the poor detection of understory fires, fires occurring under the presence of heavy smoke, agricultural fires, and false positives due to other sources (e.g., cloud shadows and deforested areas), and other land use changes such as clearing and intertidal movement, causing BA estimates to vary widely between different products^15–18^.

Other BA information is derived from a variety of sources, including, for instance, ground-based mapping and aerial photography. For several regions, such as Australia, Canada, Chile, the European Union (Europe hereinafter), and the U.S.A., national fire inventories of BA data are generally available for a longer period than the global satellite BA products^13^. Although these fire datasets cannot be considered error-free “ground truth”, they represent the longest government-sourced fire information available today at the regional scale and have been widely used as reference datasets in multiple studies^16,19–29^. Although these regional databases are publicly available, merging them together is a time-consuming task, partly because these archives are stored in different formats and resolutions, from spreadsheets of BA data over sub-regions to shapefiles with detailed information of all mapped fires. A harmonized regional fire dataset based on regional/national archives may simplify their use in intercomparison and assessment studies and could complement and temporally extend the BA products based on satellite data.

Here, we describe the creation of an integrated and open-access database composed of available BA records from government-sourced observations in Australia, Europe, Canada, Chile, and the U.S.A. This gridded BA database of national wildland fire data (ONFIRE database) aims to transform the authoritative fire data into a public, easily accessible, harmonized, and usable database to facilitate broad-scale fire research^30,31^. Owing to the format of the source inventories, the ONFIRE database resolution is constrained to a monthly, 1° grid, standardised for each area, thus reducing the likelihood of errors in the code and the effort needed to adapt it to different source formats. Although this level of aggregation may obscure the details of the variability within grids, some of the temporal and spatial noise present in the higher-resolution data may be reduced and provide a comprehensive regional-scale BA analysis. A survey of users’ needs for BA products^15^ emphasised the importance of having a coarser-scale product to reduce the data volume and processing cost. In addition, they recommended that the BA products should be available in ASCII and netCDF formats. Taking these considerations into account, the ONFIRE dataset^32^ is released in both these formats and is freely available in a Zenodo permanent repository (https://zenodo.org/record/8289245), which will ensure the usability of this information for a wide range of applications. In addition, the data are accompanied by the corresponding metadata with the original references and inventory sources. Finally, we explore the main opportunities and constraints related to the use of this dataset, as well as potential future extensions for this open-source solution. We specifically encourage any researcher/fire agency to share their data through the ONFIRE initiative.

## Methods

**Fig. 1:**
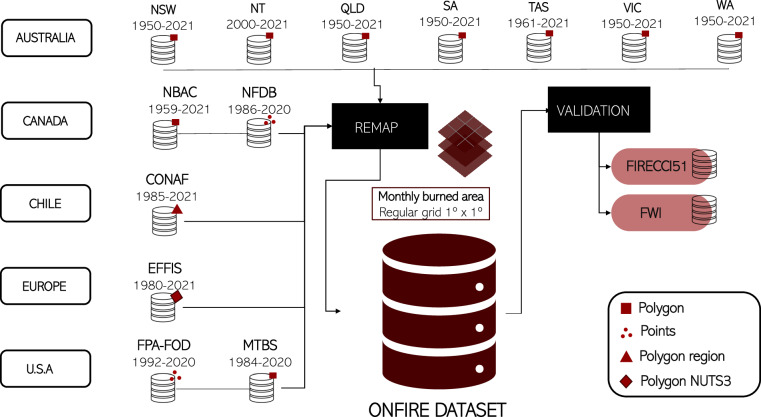
Schematic workflow for the harmonisation of BA observations from different regions to develop the ONFIRE dataset.

We compiled BA observations from government agencies in Australia, Canada, Chile, Europe, and the U.S.A. then remapped the monthly BA at a common 1° × 1° grid. Finally, we validated the ONFIRE dataset (Fig. 1). The temporal and spatial BA resolution adopted by the ONFIRE dataset resulted from the availability of Europe and Chile monthly data provided with a resolution of approximately 1°.

### Remapping methodology

The data were effectively assigned to a 1° × 1° grid (we used the 1° grid used in the Sixth Assessment Report of the United Nations Intergovernmental Panel on Climate Change^33,34^) while considering the specific conditions of the original format, i. point data (generally corresponding to fire polygon centroid), ii. fire polygons, or iii. region-level data:

I. Point data
  Assigned the BA value to the grid cell containing the point.
  Determine the month for the grid cell based on the starting date of the fire.
II. Polygon of Fire Perimeters
  Calculated the centroid for the corresponding fire polygon.
  Assigned the BA value to the grid cell containing the centroid.
  Determined the month for the grid cell based on the starting date of the fire.
III. Total BA over a Region:
  Determine the number (*n*) of gridpoints at a resolution of 0.1° within the region, to ensure relatively small regions are not excluded.
  Divided the BA value of the region by *n*.
  Assigned the resulting BA value to each 0.1° grid cell within the region, conserving total BA by distributing the BA proportionally across the grid cells within the region.
  Sum all BA values at 0.1° that fall on a grid cell of the 1° grid.

### Compilation of wildland fire data

The BA observations and full references that composed the ONFIRE datasets are derived from government agencies in Australia, Canada, Chile, Europe, and the U.S.A. (Fig. 1, Table 1). The definition of wildland fire is inconsistent across countries and time periods. These fires are commonly referred to by different names such as wildland fires, wildfires, bushfires, biomass burning, forest fires, agricultural fires, and prescribed fires^35^, in addition to some similar names in Spanish (for Chile). Among these, the most extensively documented fires in our database are categorised as “wildland fires” (“incendios forestales” for Chile). This classification encompasses planned, prescribed, or controlled burning and unplanned or accidentally ignited fires known as “wildfires”, which are considered a distinct class of wildland fires, separate from prescribed or controlled burning. It is worth noting that agricultural fires, which involve burning agricultural wastes from e.g., sugar cane in South America, rice in Asia, or wheat stubble in temperate countries (south-central Chile), can affect vast areas. However, these fires are not included in our dataset (differently from remote sensing data) as they are typically not reported in (wild) fire records^36^.

**Table 1:** List of each regional source and their available periods included in the ONFIRE dataset.

Region	Source	Available period
Australia	New South Wales (NSW): https://datasets.seed.nsw.gov.au/dataset/fire-history-wildfires-and-prescribed-burns-1e8b6	1950–2021
Australia	Northern Territory (NT): https://firenorth.org.au/nafi3/	2000–2021
Australia	Queensland (QLD): https://qldspatial.information.qld.gov.au/catalogue/custom/	1950–2021
Australia	South Australia (SA): https://data.sa.gov.au/data/dataset/fire-history	1950–2021
Australia	Tasmania (TAS): http://listdata.thelist.tas.gov.au/opendata/	1961–2021
Australia	Victoria (VIC): https://discover.data.vic.gov.au/dataset/fire-history-records-of-fires-across-victoria-showing-the-fire-scars2	1950–2021
Australia	Western Australia (WA): https://catalogue.data.wa.gov.au/dataset/dbca-fire-history	1950–2021
Canada	NBAC: https://cwfis.cfs.nrcan.gc.ca/datamart/metadata/nbac	1986–2020
Canada	NFDB: https://cwfis.cfs.nrcan.gc.ca/downloads/nfdb/fire_pnt/current_version/NFDB_point_large_fires.zip	1959–2021
Chile	CONAF: https://www.conaf.cl/incendios-forestales/incendios-forestales-en-chile/estadisticas-historicas/	1959–2021
Europe	EFFIS: https://effis.jrc.ec.europa.eu/	1980–2021 (see Table 2 for more details)
U.S.A.	MTBS: https://mtbs.gov/direct-download	1984–2020

### Australia

In Australia, the lack of a unified national database has led to the compilation of data from different fire management agencies in each state and territory that have provided statistics (Table 1) to obtain a complete picture of the area burned in the country^19,21^. Data from these agencies are available in the form of polygons in vector files that have been derived from a variety of sources, including terrestrial mapping, aerial photography, GPS boundary plotting from ground assessments, and remote sensing. Consistent burned area mapping from 2000 for north and central Australia are freely available as shapefiles and GeoTIFF files from the NAFI website^37^ managed by Charles Darwin University funded by the Australian Government for carbon accounting. These fire scar maps are generated through a semi-automated process approximately weekly, using 250 m image bands (red and near-infrared) from the Moderate Resolution Imaging Spectroradiometer (MODIS) sensor. They are collated on a monthly basis and cover a significant portion (75%) of the Australian continent (specifically, the Northern Territory, the majority of Queensland, the West Australian rangelands and savannas, and the northern half of South Australia^38,39^ We used NAFI scars for the following areas: the Northern Territory and Alice Springs, Barkly, Daly - Tiwi - West Arnhem, Darwin City, Darwin Suburbs, East Pilbara, Katherine, Kimberley, Litchfield, Outback - North, Outback - South, and Palmerston.

All these data have been extensively validated and used previously as reference products in several studies^21,40–43^. The types of fires mapped in these datasets include both prescribed fires and wildfires. Wildfires are frequently labeled “bushfires”, in the more heavily populated regions of the south-east and south-west. In databases, or for example public warning systems, “bushfires” are “unplanned fires”, or “wildfires” as opposed to “planned fires”, “hazard reduction burns” or “prescribed fires”.

### Canada

There are numerous fire databases in Canada, and they each come with a different set of limitations (Hanes *et al*., 2019). The Canadian National Fire Database (NFDB) is best for the exploration of long-term BA using fire agency-collected data^22,44–49^ and is most comparable to other national datasets over their common time period. The NFDB provides fire point data with longitude, latitude, size, month, and year of fire as provided by individual fire management agencies. Only fires greater than 200 hectares are included in the database and only data from 1959 are included in this analysis because of the data quality concerns prior to this year^50^. From 1959 to 2021, the dataset records consist of ~1.3 Mkm^2^ of BA and ~18000 occurrences for the entire country.

A second fire database has recently gained wide use in Canada: the National Burned Area Composite (NBAC) dataset^51–53^. The NBAC dataset is considered the most consistent and accurate wildfire polygon database in Canada. This fire polygon database is derived from 30-m Landsat imagery and high-quality agency imagery of spatial resolution <30 m since 1986^51^; it has been updated regularly after it was published. Because NBAC does not cover fires before 1986, one must combine NBAC with NFDB to construct the fire database when fire records from early years are required^52^. From 1986 to 2020, the dataset records consist of ~750000 km^2^ and ~35000 occurrences for the entire country.

Recent comparisons have identified that BA estimates in Canada based on NFDB, on average may be overestimated by ~20% especially for early years (i.e., before 2000) prior to greater use of remote sensing products^52^. The imprecisions are primarily attributable to the limitations of the fire perimeter mapping techniques that were accessible during that period, such as sketch mapping, GPS-based digitization, and geographic coordinate point buffering. These methods often do not consider unburned islands and water bodies inside the fire perimeter^54,55^. Acknowledging that limitation in these datasets, we provided gridded BA for both the NFDB and NBAC datasets. In summary, we introduce two datasets for Canada, namely ONFIRE-NBAC and ONFIRE-NFDB, which are named in reference to their respective original sources.

### Chile

In Chile, the official fire database is the responsibility of the Chilean Forest Service (CONAF) and comprises the period between 1985 and 2021. Although there is a historical database of the number of fires and BA since 1964, only records starting in 1985 provide a reliable monthly BA for each administrative region of the country. Fire records include the number of fires and BA considering different vegetation types (natural vegetation, forest plantations, shrublands, pastures, excluding agricultural burnings of crops and grass). From 1985 to 2022, the dataset records consist of ~28000 km^2^ of BA and ~225000 occurrences for the entire country. The CONAF dataset has been validated and used in several studies to analyse the relationships between climate (e.g., drying trends and heat waves), land use, socioeconomic factors, and wildfires^4,23–25,56–61^.

### Europe

The European Forest Fire Information System^62^ (EFFIS), established by the Joint Research Centre and the Directorate General for the Environment of the European Commission, is the primary source of harmonised fire data and provides the official fire statistics of the European Union. Via direct consultation with the European Forest Fire Information System (EFFIS), we obtained monthly data on Burned Areas at the NUTS3 territorial level, as classified by the 2016 Nomenclature of Units for Territorial Statistics, which corresponds to aggregated municipal or provincial units^63^. This dataset includes several European countries that actively share data with EFFIS, as detailed in Table 2. The EFFIS dataset consists of BA (in hectares, the totals are for fires of 1 ha or greater) occurring in forests, other wooded lands, and other non-wooded lands reported by the country, while areas designated as agricultural or other artificial surfaces are not included^62^. The dataset records consist of ~150000 km^2^ of BA and ~700000 occurrences for the entire region. This dataset had several limitations, namely the lack of fire perimeter data because the raw data are owned by the contributing countries; EFFIS is only allowed to aggregate it by NUTS and share the resulting data at the NUTS level. Another limitation is the lack of recent data from several countries. This is because the entire data provision system is voluntary without a regulated standard for contributions. In addition, some countries do not have a national scheme in place to collect and share data, so they collect individual fire data only from certain regions or forest types (e.g., nationally owned forests), or the data are collected within the country, but these data are not shared with EFFIS. This can lead to fewer fires in the database than those registered. On the other hand, some countries may add records to the database that they do not count as forest fires in national statistics (e.g., agricultural fires). Nevertheless, this database constitutes the state-of-the-art information available today in Europe and shows high agreement with satellite products over common periods^16^. Moreover, several studies have used EFFIS data to assess fire evolution in Europe^26,64^ and its drivers^65,66^.

**Table 2:** Details of the EFFIS dataset, with the starting and ending period available and possible comments.

Country	From	To	Comments
Bulgaria	2005	2019	
Cyprus	2000	2019	Only Greek part of CY
Croatia	1996	2019	
Czech Republic	2004	2019	
Estonia	2005	2020	
Finland	2005	2020	
France	1985	2020	Only Mediterranean regions before 1992
Germany	1994	2021	Some NUTS regions missing
Greece	1983	2011	Some NUTS regions missing
Hungary	2002	2020	
Italy	1985	2015	Special status or autonomous regions (e.g., Sicily, Sardinia) often missing
Lithuania	2004	2021	
Latvia	2004	2018	
Netherland	2017	2020	
Poland	1994	2020	
Portugal	1980	2020	
Romania	2004	2021	
Spain	1985	2015	Some NUTS regions missing
Slovakia	2004	2018	
Slovenia	1995	2021	
Sweden	1996	2020	

### U.S.A

The Fire Program Analysis fire-occurrence database (FPA-FOD) dataset^67^ provides information on wildfires that occurred in the U.S.A. between 1992 and 2020 and includes information on the location, size, and cause of the fire. This is the 6th Edition of the FPA-FOD dataset, first published in 2013^68^. The data were compiled from the final fire reports of federal, state, and local fire organisations and include over 2.3 million geo-referenced records, providing the most comprehensive accounting of U.S.A. wildfire activity for the time period^67,68^. While the FPA-FOD data does not impose a minimum fire size requirement, it is worth noting that agencies typically report the smallest fires in the dataset as being 0.04 hectares in size. The FPA-FOD dataset does not encompass prescribed/controlled burning, thereby failing to provide a comprehensive representation of the total burned area resulting from biomass burning, landscape fires, or even wildland fires as a whole. However, although inevitably incomplete in some aspects^68^, this dataset is widely used by researchers and practitioners to study the causes and consequences of wildfires in the U.S.A. Applications of the data include analysing the relationships between climate, land use, and wildfires, as well as developing fire management strategies to prevent and mitigate the impacts of wildfires^27,69–72^.

Another fire dataset available for the U.S.A. is the Monitoring Trends in Burn Severity (MTBS) data^73–75^. This is a semi-automated program that employs pre-fire and post-fire Landsat imagery for the generation of estimated BA severities. The MTBS dataset aims to provide comprehensive coverage of large fire events throughout the United States, subject to the criterion that fires must surpass a specific size threshold (>2.02 km^2^ and >4.05 km^2^ in the eastern and western US, respectively) since 1984 and consists of ~830000 km^2^ of BA and ~30000 occurrences for the entire country. MTBS data have been widely used by researchers to examine a variety of environmental science topics^6,29,76–78^.

In summary, we introduce two datasets for the U.S.A., namely ONFIRE-FPA-FOD, and ONFIRE-MTBS, which are named in reference to their respective original sources.

## Data Records

### Database structure

ONFIRE data are available to the public through version-controlled releases of the database on Zenodo^32^, and all codes used in the development and validation of the dataset^79^ are freely available, in line with the FAIR Guiding Principles^80^. ONFIRE is a 1° × 1° degree lat-long gridded dataset with, for each grid cell, the latitude and longitude of the center and the square metres burned per month. Each month’s data includes the total area in square metres for fires reported to start within that month. For instance, a fire that started on July 31 and burned for a month will be entirely reported in July. Data are stored in decimal degrees, GCS WGS84. The dataset contains fire estimates over the various recorded lengths, up to 2021, and spatial coverages (Table 1). The database is provided for each dataset and region in several formats: NetCDF^81^, RData^82^, and ASCII^83^. In total, we provide 21 files: 3 for each of the 5 regions, and two additional datasets are provided corresponding to the NFDB and NBAC sources for Canada, and the FPA-FOD and MTBS sources for the U.S.A.

## Technical Validation

The original records included in the database are provided by government and non-government agencies and as described in the previous section, have already been used individually in several regional studies (although not all together). We acknowledge, however, the potential limitations previously reported in national data^84,85^. These data commonly face various challenges, including periods of inaccessible or poor-quality data^36,86^, difficulties in estimating burned area from field observations^13,85^ uncertainties arising from different fire reporting protocols between countries and/or protocol changes over time^87^, and high political controls on BA statistics^84,88^. Thus, even though they cannot clearly be considered error-free “ground truth”, these data represent the main source of authoritative fire histories available today, before and during the satellite era. In addition, users can evaluate the validity and accuracy of the original source for each regional dataset from its references (Table 1). It is also worth noting that here we provide BA data at a monthly scale and over a 1° grid resolution, therefore substantially reducing any uncertainties and noise that could be present at higher temporal and spatial resolutions. In addition, we provide information on the total BA, which is primarily influenced by the extent of large fires. These large fires are typically monitored due to their significant impacts (however it is worth noting that ONFIRE BA grid cells can also be affected by fires of less than 100 hectares^89^). Also, while caution is necessary when relying solely on BA, it remains a useful tool for understanding the overall magnitude and spatial extent of fire events^90^ as it provides a quantitative measure that can be easily compared across different fire events and regions.

We also followed two different approaches to provide a quantitative assessment of the ONFIRE data: (1) we compared it with an independent satellite-based dataset, to examine the coherence among the datasets, and (2) we made use of the ONFIRE dataset to perform the same analysis of a previous study^11^, thus comparing the results. This two-step assessment process is crucial to ensure the integrity and quality of the ONFIRE data.

### Comparison with an independent dataset

We compared the monthly BA data from the ONFIRE dataset against the remotely sensed FireCCI51 data^91^, available for the period 2001–2020 at an original resolution of 250 m × 250 m. This is the most recently developed global BA dataset and complements existing global BA products using surface reflectance data from the Near Infrared (NIR) band of the MODIS instrument aboard the Terra satellite, alongside active fire detection information from the MODIS sensors on both Terra and Aqua satellites. When comparing our dataset with FireCCI51, it is important to recognize that although FireCCI51 is referenced as an independent dataset, for the Northern region of Australia, both FireCCI51 and NAFI datasets derive data from the same MODIS satellite sensor. Despite this commonality in data sources, the methodologies employed by each dataset are distinct. FireCCI51 utilizes a fully automated algorithm, whereas NAFI employs a method that combines automated analysis with visual checks and supervised classification. Consequently, despite the similarity in the input data, the significant differences in the processing approaches justify the characterization of this as an intercomparison analysis. In any case, we acknowledge a certain level of non-independence inherent in the data source, which must be carefully considered in our evaluation. A recent comparison amongst remotely sensed and inventory datasets for BA in Mediterranean Europe showed that FireCCI51 had the best agreement with EFFIS BA data overall^16^. To perform this comparison, a series of steps were undertaken. First, we downloaded the BA data at the grid resolution (0.25° × 0.25°) and we removed non-natural FireCCI51 BA data to align our analysis with official fire statistics, which typically exclude fires on agricultural land. The FireCCI51 dataset provides the sum of burned area for each land cover category. Therefore, we calculated the sum of the burned area by considering all land cover categories except for “cropland, rainfed”, “cropland, irrigated or post-flooding”, and “mosaic cropland (>50%) in line with other methodology”^16^. Then, the BA data were upscaled by summing all FireCCI51 grid points at 0.25° included in the corresponding 1° grid point of the ONFIRE dataset. Then, the mean annual aggregated BA series, calculated during the overlapping period, were compared. Finally, the temporal similarity of the monthly BA series was evaluated using Spearman correlation.

**Fig. 2:**
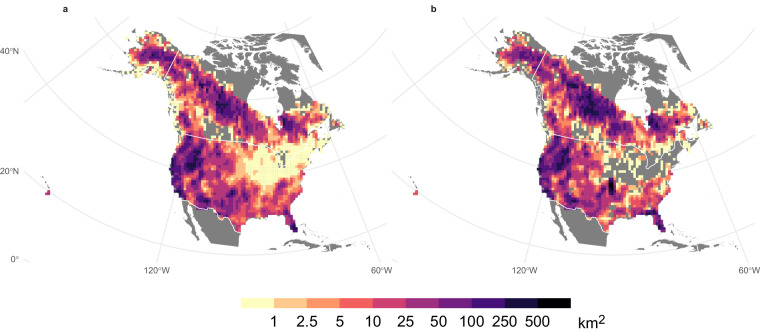
Mean annual burned area (km^2^) using data from various sources for Canada and the U.S.A. The different panels show the results obtained by combining different data sources: (**a**) ONFIRE-NBAC for Canada (1986–2020) and ONFIRE-FPA-FOD (1992–2020) for the U.S.A.; (**b**) ONFIRE-NFDB for Canada (1986–2020) and ONFIRE-MTBS (1992–2020) for the U.S.A.

**Fig. 3:**
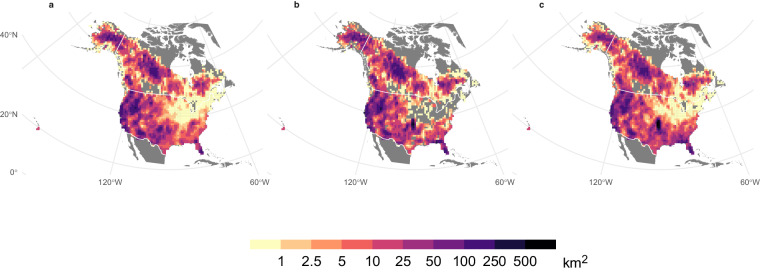
Mean annual burned area (km^2^) averaged over the period 2001–2020 using data from various sources for Canada and the U.S.A. The different panels show the results obtained by combining different data sources: (**a**) ONFIRE-NBAC for Canada and ONFIRE-FPA-FOD for the U.S.A.; (**b**) ONFIRE-NFDB for Canada and ONFIRE-MTBS for the U.S.A.; (**c**) FireCCI51. Areas with no data are shown in grey.

There is a high degree of similarity between the mean annual area burned included in ONFIRE for U.S.A. and Canada (Fig. 2a,b). Indeed, the spatial (Spearman) correlation coefficient between the two patterns is 0.89 (p-value < 0.01). The total annual burned area in Canada averaged over the period 1986–2020 was approximately 17668 km^2^ based on the ONFIRE-NBAC dataset, while the ONFIRE-NFDB dataset yielded an average value of 24125 km^2^. This represents a difference of 27% between the two datasets, which is similar to the findings of a study conducted in 2021^54^who reported a 23% difference considering fire data from 1986 to 2018. Higher similarity appears considering the annual BA in the U.S.A. averaged over the common period of 1992–2020. Specifically, the BA estimated using the ONFIRE-FPA-FOD dataset was found to be approximately 24,651 km^2^/year, and the ONFIRE-MTBS dataset yielded an estimate of around 24,744 km^2^/year, indicating a substantial agreement in the recorded values. It should be highlighted that in the northeastern United States, there are several regions without data in the ONFIRE-MTBS analysis, as indicated by the grey areas in Figs. 2b, 3b. This absence of data is likely attributable to the minimal fire activity in these regions, where the annual average burned area is generally less than 1 km², while the MTBS data in this area has a detection threshold, with the minimum identifiable burned area being over 2.02 km². The comparison of these data against FireCCI51 over the period 2001–2020 provides evidence of similarities among the datasets under examination (Fig. 3a–c). Specifically, the correlation between ONFIRE-NBAC + ONFIRE-FPA-FOD and FireCCI51 was 0.89, and between ONFIRE-NFDB + ONFIRE-MTBS and FireCCI51 was 0.89. The annual BA in Canada averaged over the common period 2001–2020, was quite similar among the three datasets: 16800, 22266, and 17525 km^2^/year for, respectively, ONFIRE-NBAC, ONFIRE-NFDB, and FireCCI51. Again, lower differences have been found over the U.S.A., with 28660, 29997, and 30552 km^2^/year for ONFIRE-FPA-FOD, ONFIRE-MTBS, and FireCCI51.

**Fig. 4:**
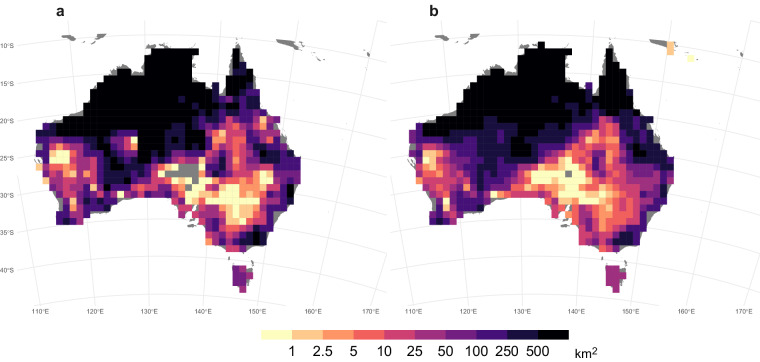
Mean annual burned area (km^2^) for the ONFIRE (panel a) and the FireCCI51 (panel b) datasets for Australia. Data for the period 2001–2020, or shorter depending on the data availability (see Tables 1, 2). Areas with no data are shown in grey.

**Fig. 5:**
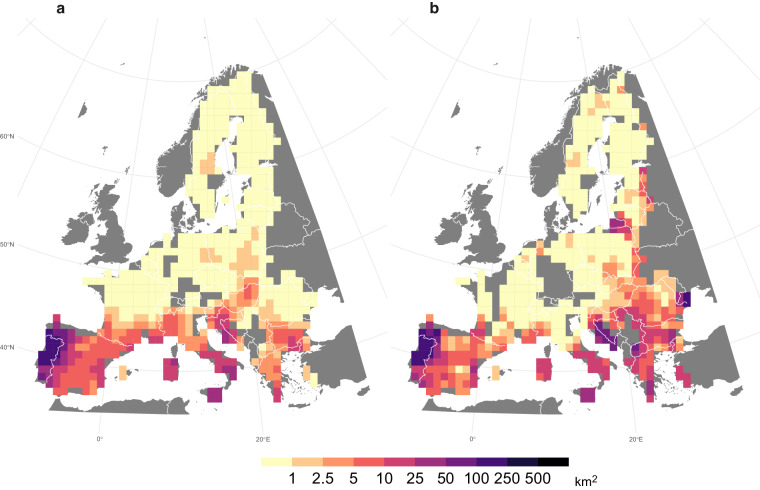
Mean annual burned area (km^2^) for the ONFIRE (panel a) and the FireCCI51 (panel b) datasets for Europe. Data for the period 2001–2020, or shorter depending on the data availability (see Tables 1, 2). Areas with no data are shown in grey.

**Fig. 6:**
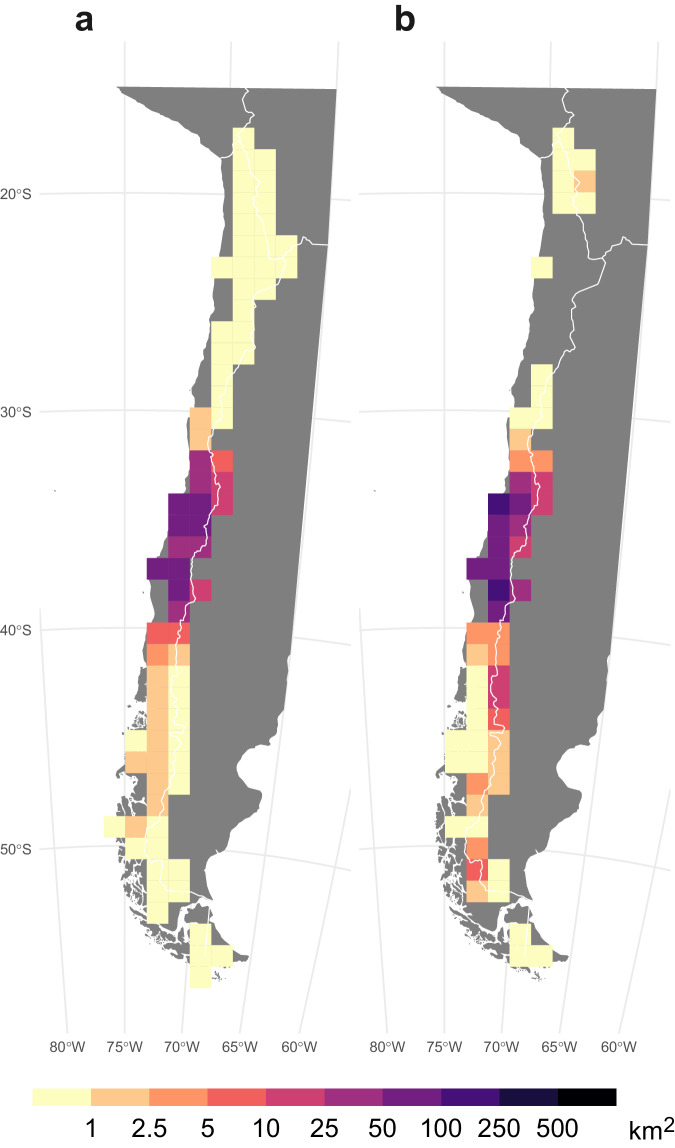
Mean annual burned area (km^2^) for the ONFIRE (panel a) and the FireCCI51 (panel b) datasets for Chile. Data for the period 2001–2020, or shorter depending on the data availability (see Tables 1, 2). Areas with no data are shown in grey.

A good agreement was also observed between the annual BA averaged over the period 2001–2020, as estimated by the ONFIRE, and the BA from FireCCI51 datasets, for Australia, Chile, and Europe (Figs. 4–6). The spatial correlation is notably high over Australia, with a coefficient of 0.87 (p-value < 0.01), and the corresponding total annual BA values for the ONFIRE and FireCCI51 datasets are 469950 km^2^/year and 431892 km^2^/year, respectively. In Chile the level of agreement between the datasets is lower, with a spatial correlation coefficient of 0.62 (p-value < 0.01), and the averaged values of total annual BA are 1027 km^2^/year and 1848 km^2^/year for the ONFIRE and FireCCI51 datasets, respectively. In Europe, the spatial agreement is lower, with a correlation coefficient of 0.59 (p-value < 0.01), but the datasets provide similar estimated mean values, with the ONFIRE and FireCCI51 datasets yielding 3323 km^2^/year and 3498 km^2^/year, respectively of total annual BA. These differences may be attributed to various factors, including misinterpretations (remote sensing detects fires that are not fires), or possibly underreporting in national statistics. However, further investigation is required to gain a comprehensive understanding of these differences, which is beyond the scope of our analysis. Our primary goal here is to present these datasets in a standardised format, facilitating similar assessments in the future. In any case, these differences emphasise the importance of cautiously considering the underlying data sources and methodologies during the conduction of analyses with fire data. It also highlights the requirement for ongoing efforts to enhance data collection and standardisation practices.

**Fig. 7:**
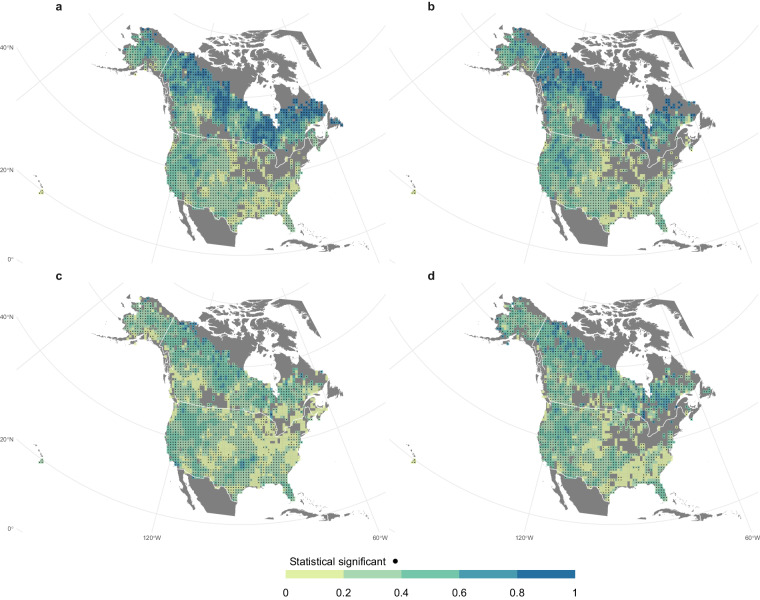
Spearman correlations between monthly estimates of burned area for the Canadian and U.S.A. regions, for different datasets and periods: (**a**) ONFIRE-NBAC against ONFIRE-NFDB in Canada for the period 1986–2020, and ONFIRE-FPA-FOD against ONFIRE-MTBS in the U.S.A. for the period 1992–2020; (**b**) same as in (**a**) but for the more recent period 2001–2020; (**c**) ONFIRE-NBAC (Canada) and ONFIRE-FPA-FOD (U.S.A.) against FireCCI51; and (**d**) ONFIRE-NFDB (Canada) and ONFIRE-MTBS (U.S.A.) against FireCCI51 Study period in (**b**), (**c**), and (**d**) is 2001–2020. Areas with no data are shown in grey.

**Fig. 8:**
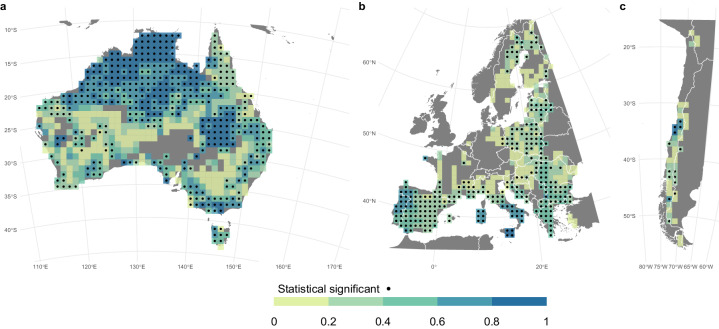
Spearman correlation between monthly burned area estimates from the ONFIRE and the FireCCI51 datasets for Australia (**a**), Chile (**b**), and Europe (**c**), during the period 2001–2020 (or shorter, depending on the data availability; see Tables 1, 2 and Fig. 1). Areas with no data are shown in grey.

In addition, a correlation analysis was performed to measure the temporal similarity among the monthly burned area estimated by the different databases (Figs. 7, 8). A consistent positive significant correlation was found for the Canada region comparing ONFIRE-NBAC and ONFIRE-NFDB, and for the USA, comparing ONFIRE-FPA-FOD and ONFIRE-MTBS, both for the largest common periods and considering the 2001–2020 periods (Fig. 7a,b). The correlations between these data and the FireCCI51 are generally significant but lower. (Fig. 7c,d).

ONFIRE and FireCCI51 data are also correlated for Australia, Chile, and Europe (Fig. 8). For the Australian region (Fig. 8a), a significant and positive correlation is observed almost everywhere, particularly in the north. It is important to note that this correlation may be partially influenced by the fact that both the ONFIRE and FireCCI51 datasets for northern territory are based on the same MODIS sensor (see the section “Technical Validation” for further details). That is, this shared data source can contribute to the observed agreement. For the Chile region (Fig. 8c), a highly significant correlation is restricted to areas where surface fire activity has been recorded in both datasets, indicating the strength and direction of the relationship of the burned area between ONFIRE and FireCCI51 is significant in these areas. In the European region (Fig. 8b), a significant and high correlation is observed where a high burned area is observed, especially in the Mediterranean and Eastern Europe (compare Fig. 5a,b, and Fig. 8b). Unlike the Chilean region, in Europe, even in areas with relatively low burned area values (such as Scandinavia), high correlations can be found.

### Reproduction of earlier published results

To assess the accuracy of ONFIRE data over a longer period, including years before the MODIS-era (early 2000s) we follow a similar approach inspired by a recent study^92^, which validated historical yields for major crops with climate data. The Canadian Fire Weather Index (FWI^93^), a widely used index to assess the meteorological fire danger worldwide, exhibits a correlation with BA over most of the globe^11,94^. We extracted the FWI^95^ data for the period 1979–2021 and available from the Copernicus Emergency Management Service^96^. In order to ensure consistency between the spatial resolution of the different datasets, the FWI data are also remapped from their original resolution (0.25° × 0.25°) to the 1° × 1° fire grid with a bilinear interpolation (using Climate Data Operators, CDO^97^). Then, following a similar methodology employed in the study of Jones *et al*.^11^, we calculate Spearman’s rank-order coefficient. The Spearman correlation coefficient was chosen due to the exponential relationship between climate (FWI) and fires (ONFIRE).

This analysis is not intended to investigate the drivers of fires or discuss the importance of the FWI but to indirectly assess the ONFIRE datasets patterns using independent climate data that prior studies have shown to be related to fire variability. Despite FWI being the most commonly applied index for meteorological rating fire danger worldwide^70,98,99^, it may not be the best predictor for explaining BA variability^100,101^. Previous studies^11,94,100,102^ demonstrated that the connection between BA and FWI varies depending on the geographical location. The relationship is stronger in regions with high biomass, where the main constraint to fire is fuel moisture, rather than fuel availability. In contrast, BA shows less sensitivity to FWI in xeric grasslands and shrublands since these systems are more constrained by fuel productivity^11^. Jones *et al*.^11^ have shown that there is a positive and statistically significant correlation between monthly BA and monthly FWI in most regions of the world. Specifically, this relationship is particularly strong in Canadian and Alaskan forests, central Chile, the Mediterranean, north and southeast Australia, and the western U.S.A. Very similar results have been found here, as we detail in the following.

**Fig. 9:**
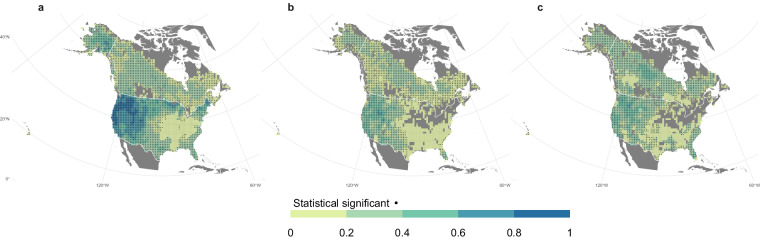
Spearman correlation between monthly Fire Weather Index (FWI) and burned area (BA) as estimated by different datasets and periods: (**a**) ONFIRE-NBAC (1986–2020; Canada) and ONFIRE-FPA-FOD (U.S.A. 1992–2020); (**b**) ONFIRE-NFDB (1979–2021; Canada) and ONFIRE-MTBS (1984–2020; U.S.A.); (**c**) FireCCI51 (2001–2020). Areas with no data are shown in grey.

**Fig. 10:**
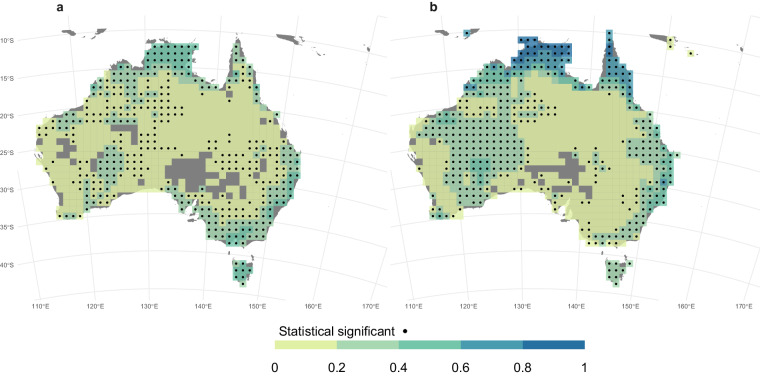
Spearman correlation between monthly Fire Weather Index (FWI) and burned area (BA) in Australia, panel (**a**) for the period 1979–2021 (or shorter, Tables 1, 2) with ONFIRE data, and panel (**b**) for the 2001–2020 period with FireCCI51 data. Areas with no data or non-significant (p-value < 0.05) correlations are shown in grey.

**Fig. 11:**
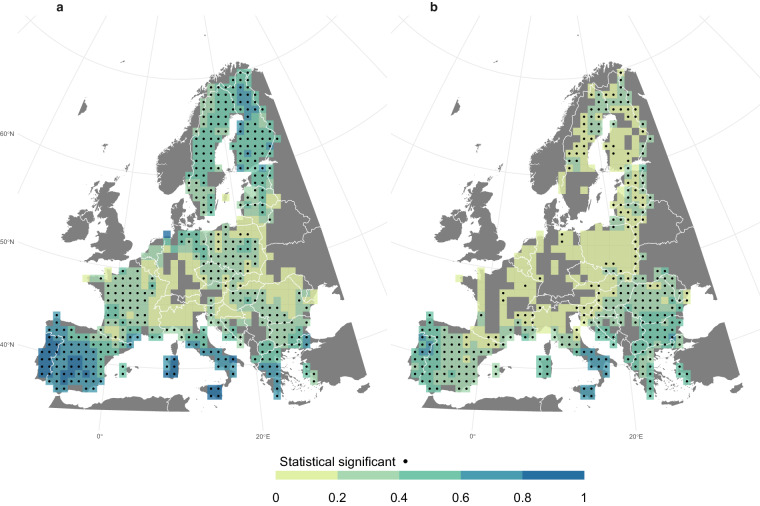
Spearman correlation between monthly Fire Weather Index (FWI) and burned area (BA) in Europe, panel (**a**) for the period 1979–2021 with ONFIRE data, and panel (**b**) for the 2001–2020 period with FireCCI51 data. Areas with no data or non-significant (p-value < 0.05) correlations are shown in grey.

**Fig. 12:**
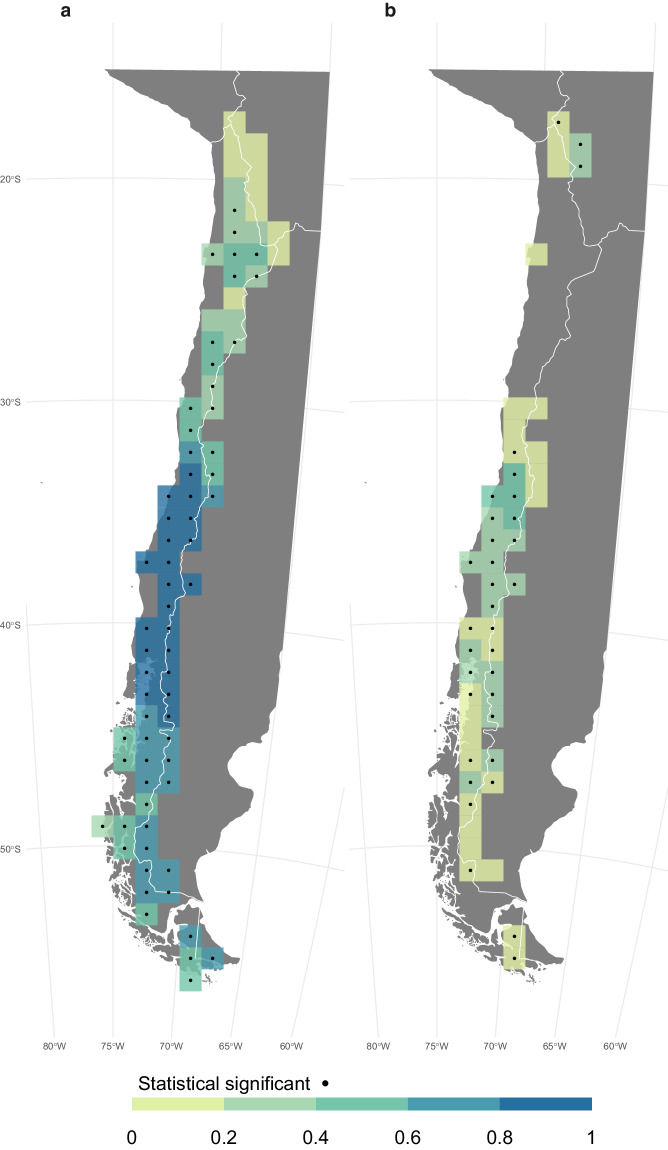
Spearman correlation between monthly Fire Weather Index (FWI) and burned area (BA) in Chile, panel (**a**) for the period 1979–2021 with ONFIRE data, and panel (**b**) for the 2001–2020 period with FireCCI51 data. Areas with no data or non-significant (p-value < 0.05) correlations are shown in grey.

The BA provided by the ONFIRE datasets and the FWI at the monthly scale (for the period 1979–2021 or shorter, depending on the data availability; see Tables 1, 2) are well correlated (Figures ranging from 9 to 12) and mostly resemble the assessment shown by Jones *et al*.^11^. For the USA region, higher correlations over the western U.S.A. (of more than 0.75) are observed considering the ONFIRE-FPA-FOD (Fig. 9a) than considering the ONFIRE-MTBS data (Fig. 9b). Interestingly, Fig. 9c shows that FireCCI51 and ONFIRE data are on the same order of magnitude of correlations compared to FWI, except over the western U.S.A., where ONFIRE-FPA-FOD shows higher values. For Australia (Fig. 10a), a significant correlation between burned area (ONFIRE) and FWI data is shown in the north and southeast of the region. This may be explained as the limited availability of fuel plays a crucial role in shaping fire patterns in inland Australia. Conversely, in the forested regions of the eastern and southwestern coastlines, the dryness of the existing fuel further exacerbates the fire risk. Significant correlations are found in the European region (Fig. 11a), with particularly high values over the Iberian Peninsula, southern France, and Italy. In Chile (Fig. 12a), the ONFIRE data show a significant and high correlation practically throughout the region, with higher values in the central part of the country. In general, we have observed similar patterns when analysing the FireCCI51 data, albeit with slightly lower correlation values (Figs. 10b, 11b, 12b).

## Usage Notes

### Informed user

The use of the ONFIRE dataset comes with some caveats and users should be aware of the limitation of these data. While the original data that form this dataset represent the most comprehensive fire inventories in each considered region, these are not free from uncertainties and are not fully complete. Similar to other fire databases^13,64,72,103^, small fires are probably frequently missing and the minimum burned area for which a fire is recorded may not be consistent over the study period (as also noticed in other subregional datasets^87^. Nevertheless, since BA is generally dominated by large fires, the exclusion or inclusion of small fires (e.g., <1 ha) is not expected to significantly affect the total BA in most regions^87^. Also, it is important to note that in the ONFIRE dataset, certain datasets such as the MTBS and NFDB have inclusion thresholds for fires larger than 200 ha and 400 ha, respectively. While these thresholds exclude smaller fires, our comparative analysis across different datasets for northern America suggests that the exclusion of fires below these thresholds does not significantly impact the total BA estimations. This observation aligns with the general understanding that BA is predominantly influenced by larger fires, that are usually recorded, presumably because of their bigger impacts. The results presented here indicate that ONFIRE can be regarded as a reliable source of standardised information on historical fire activity, despite its inherent limitations. One notable advantage of ONFIRE is its ability to cover the longest period of monthly data compared to other available, and widely used, datasets in the scientific community. This addresses a longstanding request from the fire science community^15^ and for the benchmarking of fire-enabled global vegetation models^104^. Additionally, ONFIRE complements existing databases^36^ which only provide yearly data until 2000, and the FireCCILT11 dataset^105^ which covers the period from 1982 to 2018 but is cautioned against applying in time series fire analyses^106^. Another strength of ONFIRE lies in its focus on wildland fires. The individual datasets constituting ONFIRE are curated to primarily include fires in natural landscapes, while fires over agricultural or other artificial surfaces are deliberately not included in the source data sets. This focus underscores the utility of ONFIRE in emphasising climate and vegetation-related fires, making it particularly valuable for assessments and projections of natural fire regimes.

In any case, we recommend that end-users utilise the ONFIRE data, in tandem with other fire datasets, when available, to conduct a more thorough analysis. The growing awareness of the inherent limitation in earth observation products now relies mainly on uncertainty assessments obtained by comparing existing datasets^107,108^. Consequently, users are encouraged to contemplate the above limits when planning analysis based on ONFIRE. That is, fire datasets may have different levels of accuracy, coverage, and temporal resolution, so it is essential to evaluate their suitability for the specific research purpose carefully and to compare them with other available datasets when possible. An important aspect to take into account is spatial/temporal resolution. If the goal is, for instance, to study the relationship between climate and burned area, a grid resolution of 1° would likely be sufficient, as it provides a good balance between spatial resolution and computational efficiency. However, studies on specific regions or land use types may require a higher-resolution grid.

### Nature of a living dataset

Finally, we would like to acknowledge that the data contained within the ONFIRE dataset embody the collective work of fire mapping, rescue, and maintenance and required an enormous amount of time, effort, and resources to generate. ONFIRE has harmonised all these different datasets but does not claim their generation. When reusing (and when realistically feasible), we advise users to cite both ONFIRE and references to the original data to ensure proper attribution. Owing to the proven utility of the regional data source that composed this dataset, a specific common repository for these data may be of significance to the research community. This database will increase the reuse of this curated information for various applications. Version-controlled releases of the dataset, additional references, and metadata can be found on Zenodo (https://zenodo.org/record/8289245). Despite our best efforts to collect authoritative data and assess them in a common framework, it may be possible that we have overlooked errors in the data sets. We strongly encourage the users to report any issues or omissions by contacting the authors. Our intention is that any erroneous information will be updated in future releases of the dataset. Likewise, we encourage the community to contribute new data for other regions of the world so that the database can continue to grow, with the hope that ONFIRE will remain a useful resource for years to come.

## Data Availability

All codes used in the development and validation of the ONFIRE Dataset^32^ are freely available on Zenodo^79^: https://zenodo.org/records/10512198.

## References

[1] Pausas JG, Keeley JE (2019). Wildfires as an ecosystem service. Frontiers in Ecology and the Environment.

[2] Pyne S (2010). The Ecology of Fire. Nature Education Knowledge.

[3] Senande-Rivera M, Insua-Costa D, Miguez-Macho G (2022). Spatial and temporal expansion of global wildland fire activity in response to climate change. Nat Commun.

[4] Bowman D (2018). Wildfire science is at a loss for comprehensive data. Nature.

[5] Pausas JG, Keeley JE (2021). Wildfires and global change. Frontiers in Ecology and the Environment.

[6] Abatzoglou JT, Williams AP (2016). Impact of anthropogenic climate change on wildfire across western US forests. Proceedings of the National Academy of Sciences.

[7] Bilbao, B. *et al*. Incendios forestales. En: Adaptación frente a los riesgos del cambio climático en los países iberoamericanos – Informe RIOCCADAPT. in 459–524 (2020).

[8] Fernandez-Anez N (2021). Current Wildland Fire Patterns and Challenges in Europe: A Synthesis of National Perspectives. Air, Soil and Water Research.

[9] Forkel M (2019). Emergent relationships with respect to burned area in global satellite observations and fire-enabled vegetation models. Biogeosciences.

[10] Chuvieco E (2021). Human and climate drivers of global biomass burning variability. Science of The Total Environment.

[11] Jones MW (2022). Global and Regional Trends and Drivers of Fire Under Climate Change. Reviews of Geophysics.

[12] Zheng B (2023). Record-high CO2 emissions from boreal fires in 2021. Science.

[13] Short KC (2015). Sources and implications of bias and uncertainty in a century of US wildfire activity data. Int. J. Wildland Fire.

[14] Chuvieco E (2019). Historical background and current developments for mapping burned area from satellite Earth observation. Remote Sensing of Environment.

[15] Mouillot F (2014). Ten years of global burned area products from spaceborne remote sensing—A review: Analysis of user needs and recommendations for future developments. International Journal of Applied Earth Observation and Geoinformation.

[16] Turco M, Herrera S, Tourigny E, Chuvieco E, Provenzale A (2019). A comparison of remotely-sensed and inventory datasets for burned area in Mediterranean Europe. International Journal of Applied Earth Observation and Geoinformation.

[17] Campagnolo ML, Libonati R, Rodrigues JA, Pereira JMC (2021). A comprehensive characterization of MODIS daily burned area mapping accuracy across fire sizes in tropical savannas. Remote Sensing of Environment.

[18] Rodrigues JA (2019). How well do global burned area products represent fire patterns in the Brazilian Savannas biome? An accuracy assessment of the MCD64 collections. International Journal of Applied Earth Observation and Geoinformation.

[19] Bowman DMJS (2020). Vegetation fires in the Anthropocene. Nat Rev Earth Environ.

[20] Royé D (2020). Wildfire burnt area patterns and trends in Western Mediterranean Europe via the application of a concentration index. Land Degradation & Development.

[21] Canadell JG (2021). Multi-decadal increase of forest burned area in Australia is linked to climate change. Nat Commun.

[22] Wang X (2020). Projected changes in fire size from daily spread potential in Canada over the 21st century. Environ. Res. Lett..

[23] McWethy DB (2018). Landscape drivers of recent fire activity (2001–2017) in south-central Chile. PLOS ONE.

[24] Pozo RA, Galleguillos M, González ME, Vásquez F, Arriagada R (2022). Assessing the socio-economic and land-cover drivers of wildfire activity and its spatiotemporal distribution in south-central Chile. Science of The Total Environment.

[25] González ME, Gómez-González S, Lara A, Garreaud R, Díaz-Hormazábal I (2018). The 2010–2015 Megadrought and its influence on the fire regime in central and south-central Chile. Ecosphere.

[26] Doerr SH, Santín C (2016). Global trends in wildfire and its impacts: perceptions versus realities in a changing world. Philosophical Transactions of the Royal Society B: Biological Sciences.

[27] McLauchlan KK (2020). Fire as a fundamental ecological process: Research advances and frontiers. Journal of Ecology.

[28] Pinto MM, Libonati R, Trigo RM, Trigo IF, DaCamara CC (2020). A deep learning approach for mapping and dating burned areas using temporal sequences of satellite images. ISPRS Journal of Photogrammetry and Remote Sensing.

[29] Alizadeh MR (2021). Warming enabled upslope advance in western US forest fires. Proceedings of the National Academy of Sciences.

[30] Goldammer, J. G. *GLOBAL FOREST FIRE ASSESSMENT 1990–2000 - FRA WP 55*. (2001).

[31] Ambrosia, V. G., San Miguel-Ayanz, J., Boschetti, L., Giglio, L. & Field, R. D. The Group on Earth Observation (GEO) Global Wildfire Information System (GEO-GWIS). **2019**, NH12A-02 (2019).

[32] Gincheva A, Turco M (2023). Zenodo.

[33] Sixth Assessment Report — IPCC. https://www.ipcc.ch/assessment-report/ar6/.

[34] ATLAS/reference-grids at main · SantanderMetGroup/ATLAS. *GitHub* https://github.com/SantanderMetGroup/ATLAS/tree/main/reference-grids.

[35] Kobziar LN (2014). Fire on Earth: An Introduction. 2014. By A.C. Scott, D.M.J.S. Bowman, W.J. Bond, S. J. Pyne, and M.E. Alexander. Wiley Blackwell, Hoboken, New Jersey, USA 434 pages. Paperback, US$89.95; hardcover, US$149.95. ISBN 978-1-119-95356-2. fire ecol.

[36] Mouillot F, Field CB (2005). Fire history and the global carbon budget: a 1° × 1° fire history reconstruction for the 20th century. Global Change Biology.

[37] Northern Australian Fire Information. https://www.firenorth.org.au/nafi3/.

[38] Fisher, R. Fire extent mapping: procedures, validation and website application. in (2015).

[39] Jacklyn, P. *Extending NAFI Fire History Mapping*. (2017).

[40] Russell-Smith, J. *et al*. Bushfires ‘down under’: Patterns and implications of contemporary Australian landscape burning. *International Journal of Wildland Fire* **16**, (2007).

[41] Reynolds S (2013). Flammable Australia. Fire Regimes, Biodiversity and Ecosystems in a Changing World [Book Review]. Northern Territory Naturalist.

[42] Murphy B (2013). Fire regimes of Australia: A pyrogeographic model system. Journal of Biogeography.

[43] Lindenmayer DB, Taylor C (2020). New spatial analyses of Australian wildfires highlight the need for new fire, resource, and conservation policies. Proceedings of the National Academy of Sciences.

[44] Amiro BD (2001). Direct carbon emissions from Canadian forest fires, 1959–1999. Can. J. For. Res..

[45] Stocks, B. J. *et al*. Large forest fires in Canada, 1959–1997. **108**, **D1**, FFR5, 1–12 (2003).

[46] Flannigan M, Logan K, Amiro B, Skinner W, Stocks B (2005). Future Area Burned in Canada. Climatic Change.

[47] Parisien M-A (2006). Spatial patterns of forest fires in Canada, 1980–1999. International Journal of Wildland Fire.

[48] Boulanger Y, Parisien M-A, Wang X (2018). Model-specification uncertainty in future area burned by wildfires in Canada. International journal of wildland fire.

[49] Wang X (2017). Projected changes in daily fire spread across Canada over the next century. Environ. Res. Lett..

[50] Hanes, C. C. *et al*. Fire-regime changes in Canada over the last half century. **49**, 256–269 (2019).

[51] Hall, R. J. *et al*. Generating annual estimates of forest fire disturbance in Canada: the National Burned Area Composite. **2020**, 878–891 (2020).

[52] Skakun R, Whitman E, Little JM, Parisien M-A (2021). Area burned adjustments to historical wildland fires in Canada. Environ. Res. Lett..

[53] Skakun R (2022). Extending the National Burned Area Composite Time Series of Wildfires in Canada. Remote Sensing.

[54] Kolden CA, Lutz JA, Key CH, Kane JT, van Wagtendonk JW (2012). Mapped versus actual burned area within wildfire perimeters: Characterizing the unburned. Forest Ecology and Management.

[55] Meddens A, Kolden C, Lutz J (2016). Detecting unburned areas within wildfire perimeters using Landsat and ancillary data across the northwestern United States. Remote Sensing of Environment.

[56] Urrutia‐Jalabert. Climate variability and forest fires in central and south‐central Chile - Urrutia‐Jalabert - 2018 - Ecosphere - Wiley Online Library. https://esajournals.onlinelibrary.wiley.com/doi/full/10.1002/ecs2.2171 (2018).

[57] de la Barrera F, Barraza F, Favier P, Ruiz V, Quense J (2018). Megafires in Chile 2017: Monitoring multiscale environmental impacts of burned ecosystems. Science of The Total Environment.

[58] Gómez-González S (2019). Temperature and agriculture are largely associated with fire activity in Central Chile across different temporal periods. Forest Ecology and Management.

[59] Úbeda X, Sarricolea P (2016). Wildfires in Chile: A review. Global and Planetary Change.

[60] Miranda A (2020). Evidence-based mapping of the wildland-urban interface to better identify human communities threatened by wildfires. Environ. Res. Lett..

[61] Sarricolea P (2020). Recent wildfires in Central Chile: Detecting links between burned areas and population exposure in the wildland urban interface. Science of The Total Environment.

[62] San-Miguel-Ayanz, J. *et al*. Comprehensive Monitoring of Wildfires in Europe: The European Forest Fire Information System (EFFIS). in *Approaches to Managing Disaster - Assessing Hazards, Emergencies and Disaster Impacts*. 10.5772/28441 (IntechOpen, 2012).

[63] Overview - NUTS - Nomenclature of territorial units for statistics - Eurostat. https://ec.europa.eu/eurostat/web/nuts/.

[64] Turco M (2016). Decreasing Fires in Mediterranean Europe. PLOS ONE.

[65] Urbieta IR (2015). Fire activity as a function of fire–weather seasonal severity and antecedent climate across spatial scales in southern Europe and Pacific western USA. Environ. Res. Lett..

[66] Turco M (2018). Skilful forecasting of global fire activity using seasonal climate predictions. Nature Communications.

[67] Short, K. C. Spatial wildfire occurrence data for the United States, 1992–2018 [FPA_FOD_20210617] (5th Edition). 10.2737/RDS-2013-0009.5.

[68] Short KC (2014). A spatial database of wildfires in the United States, 1992–2011. Earth System Science Data.

[69] Calkin DE, Thompson MP, Finney MA (2015). Negative consequences of positive feedbacks in US wildfire management. Forest Ecosystems.

[70] Jolly WM (2015). Climate-induced variations in global wildfire danger from 1979 to 2013. Nat Commun.

[71] Balch JK (2017). Human-started wildfires expand the fire niche across the United States. Proceedings of the National Academy of Sciences.

[72] Syphard AD, Keeley JE, Pfaff AH, Ferschweiler K (2017). Human presence diminishes the importance of climate in driving fire activity across the United States. Proceedings of the National Academy of Sciences.

[73] Home | MTBS. https://www.mtbs.gov/.

[74] Eidenshink, J. *et al*. A Project for Monitoring Trends in Burn Severity. *Fire Ecology* **3**, (2007).

[75] Picotte J (2020). Changes to the Monitoring Trends in Burn Severity program mapping production procedures and data products. Fire Ecology.

[76] Publications | MTBS. https://www.mtbs.gov/publications.

[77] Large wildfire trends in the western United States, 1984–2011 - Dennison - 2014 - Geophysical Research Letters - Wiley Online Library. https://agupubs.onlinelibrary.wiley.com/doi/10.1002/2014GL059576.

[78] Crockett J, Westerling A (2018). Greater Temperature and Precipitation Extremes Intensify Western U.S. Droughts, Wildfire Severity, and Sierra Nevada Tree Mortality. Journal of Climate.

[79] Gincheva A, Royé D, Torres Vázquez MÁ, Turco M (2024). Zenodo.

[80] The FAIR Guiding Principles for scientific data management and stewardship | Scientific Data. https://www.nature.com/articles/sdata201618. 10.1038/sdata.2016.18 PMC4792175 26978244

[81] Unidata | NetCDF. https://www.unidata.ucar.edu/software/netcdf/.

[82] Grolemund, G. D. *Loading and Saving Data in R | Hands-On Programming with R.*

[83] Comma-separated values. *Wikipedia* (2024).

[84] Belhadj-Khedher C (2018). A Revised Historical Fire Regime Analysis in Tunisia (1985–2010) from a Critical Analysis of the National Fire Database and Remote Sensing. Forests.

[85] Pereira M, Malamud B, Trigo R, Alves P (2011). The History and Characteristics of the 1980–2005 Portuguese Rural Fire Database. Nat. Hazards Earth Syst. Sci.

[86] Koutsias N (2013). On the relationships between forest fires and weather conditions in Greece from long-term national observations (1894–2010). International Journal of Wildland Fire.

[87] Turco M, Llasat MC, Tudela A, Castro X, Provenzale A (2013). Brief communication Decreasing fires in a Mediterranean region (1970&ndash;2010, NE Spain). Natural Hazards and Earth System Sciences.

[88] *Fire, Climate Change, and Carbon Cycling in the Boreal Forest*. vol. 138 (Springer, New York, NY, 2000).

[89] Ramo R (2021). African burned area and fire carbon emissions are strongly impacted by small fires undetected by coarse resolution satellite data. Proceedings of the National Academy of Sciences.

[90] Resco de Dios V, Yao Y, Cunill Camprubí À, Boer MM (2022). Fire activity as measured by burned area reveals weak effects of ENSO in China. Nat Commun.

[91] Lizundia-Loiola J, Otón G, Ramo R, Chuvieco E (2020). A spatio-temporal active-fire clustering approach for global burned area mapping at 250 m from MODIS data. Remote Sensing of Environment.

[92] Iizumi T, Sakai T (2020). The global dataset of historical yields for major crops 1981–2016. Sci Data.

[93] Van Wagner, C. E. *Development and Structure of the Canadian Forest Fire Weather Index System*. vol. 35 (1987).

[94] Bedia J (2015). Global patterns in the sensitivity of burned area to fire-weather: Implications for climate change. Agricultural and Forest Meteorology.

[95] Vitolo C (2020). ERA5-based global meteorological wildfire danger maps. Sci Data.

[96] Fire danger indices historical data from the Copernicus Emergency Management Service (Deprecated 2023-06-14). https://cds.climate.copernicus.eu/cdsapp#!/dataset/cems-fire-historical?tab=overview.

[97] Schulzweida U (2019). Zenodo.

[98] Flannigan M (2013). Global wildland fire season severity in the 21st century. Forest Ecology and Management.

[99] Field RD (2015). Development of a Global Fire Weather Database. Natural Hazards and Earth System Sciences.

[100] Abatzoglou, J. T., Williams, A. P., Boschetti, L., Zubkova, M. & Kolden, C. A. Global patterns of interannual climate-fire relationships. *Global Change Biology*10.1111/gcb.14405 (2018). 10.1111/gcb.14405 PMC7134822 30047195

[101] Boer MM, Dios VRD, Stefaniak EZ, Bradstock RA (2021). A hydroclimatic model for the distribution of fire on Earth. Environ. Res. Commun..

[102] Silva PS, Bastos A, Libonati R, Rodrigues JA, DaCamara CC (2019). Impacts of the 1.5 °C global warming target on future burned area in the Brazilian Cerrado. Forest Ecology and Management.

[103] Westerling A (2016). Increasing western US forest wildfire activity: Sensitivity to changes in the timing of spring. Philosophical Transactions of the Royal Society B: Biological Sciences.

[104] Hantson S (2016). The status and challenge of global fire modelling. Biogeosciences.

[105] Otón G, Pereira JMC, Silva JMN, Chuvieco E (2021). Analysis of Trends in the FireCCI Global Long Term Burned Area Product (1982–2018). Fire.

[106] Giglio L, Roy DP (2022). Assessment of satellite orbit-drift artifacts in the long-term AVHRR FireCCILT11 global burned area data set. Science of Remote Sensing.

[107] Terrestrial ecosystems from space: a review of earth observation products for macroecology applications - Pfeifer - 2012 - Global Ecology and Biogeography - Wiley Online Library. https://onlinelibrary.wiley.com/doi/full/10.1111/j.1466-8238.2011.00712.x.

[108] Turco M (2020). DROP: A Probabilistic Drought Monitoring Tool. Bulletin of the American Meteorological Society.

